# Assessment of DLPNO-MP2 Approximations in Double-Hybrid DFT

**DOI:** 10.1021/acs.jctc.3c00896

**Published:** 2023-10-20

**Authors:** Hagen Neugebauer, Peter Pinski, Stefan Grimme, Frank Neese, Markus Bursch

**Affiliations:** † Mulliken Center for Theoretical Chemistry, Clausius Institute for Physical and Theoretical Chemistry, University of Bonn, Beringstraße 4, D-53115 Bonn, Germany; ‡ HQS Quantum Simulations GmbH, Rintheimer Straße 23, D-76131 Karlsruhe, Germany; ¶ Max-Planck-Institut für Kohlenforschung, Kaiser-Wilhelm-Platz 1, D-45470 Mülheim an der Ruhr, Germany

## Abstract

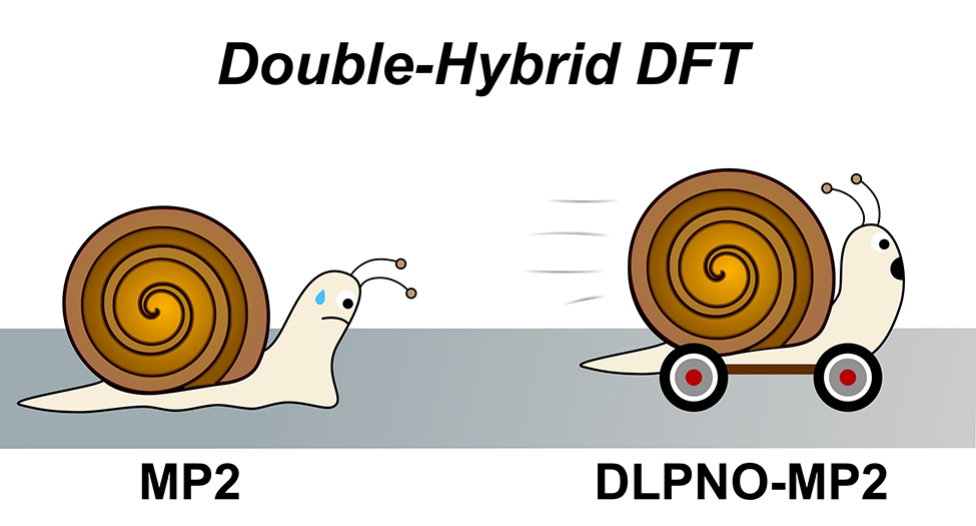

The unfavorable scaling (*N*^5^) of the conventional second-order Møller–Plesset theory (MP2) typically prevents the application of double-hybrid (DH) density functionals to large systems with more than 100 atoms. A prominent approach to reduce the computational demand of electron correlation methods is the domain-based local pair natural orbital (DLPNO) approximation that is successfully used in the framework of DLPNO-CCSD(T). Its extension to MP2 [Pinski P.; Riplinger, C.; Valeev, E. F.; Neese, F. *J. Chem. Phys.***2015**, *143*, 034108.10.1063/1.492687926203015] paved the way for DLPNO-based DH (DLPNO-DH) methods. In this work, we assess the accuracy of the DLPNO-DH approximation compared to conventional DHs on a large number of 7925 data points for thermochemistry and 239 data points for structural features, including main-group and transition-metal systems. It is shown that DLPNO-DH-DFT can be applied successfully to perform energy calculations and geometry optimizations for large molecules at a drastically reduced computational cost. Furthermore, PNO space extrapolation is shown to be applicable, similar to its DLPNO-CCSD(T) counterpart, to reduce the remaining error.

## 1 Introduction

Kohn–Sham density functional theory (DFT) is widely considered the workhorse of modern computational chemistry. Within the zoo of density functionals available, double-hybrid (DH) functionals typically represent the most accurate approaches.^1−4^ The most common DH functionals employ an admixture of the correlation energy with a fraction *a*_*C*_ computed using second-order perturbation theory (PT2) into the correlation energy expression of the respective density functional (eq 1) according to
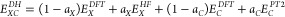
1

One of the first and most prominent DH functionals is Grimme’s B2PLYP functional^5^ that employs a 27% (*a*_*C*_ = 0.27) admixture of second-order Møller–Plesset perturbation theory (MP2) correlation energy and 53% (*a*_*X*_ = 0.53) of “exact” Hartree–Fock exchange (HFX).

A critical downside of the MP2-based DH approach is its comparably high computational demand as common MP2 formally scales with  of the system size. Accordingly, approaches to reduce the computational cost of the MP2 part of the DH calculation without losing significant accuracy are desirable. Local wave function-based correlation methods have proven highly successful in this respect. They exploit the spatial locality of electron correlation by truncation of the virtual orbital space, thus drastically reducing the number of considered orbitals. The most prominent representative of this class is the domain-based local pair natural orbital (DLPNO) approach that is frequently used in the framework of coupled cluster calculations (e.g., DLPNO-CCSD(T)).^6−10^ The DLPNO approach can also be applied to MP2 calculations which renders DLPNO-MP2 a promising candidate to use in the context of DH-DFT.^11^ The resulting DLPNO-DH scheme is available for energies, geometric gradients for closed-shell systems,^12^ polarizabilities, and NMR shieldings.^13^

The efficiency of local methods in the context of DHs has already been demonstrated for main-group thermochemistry for localized pair natural orbitals in combination with F12 explicit correlation by Mehta and Martin.^14^ But thorough studies for DLPNO-DHs that investigate the chemical space beyond the GMTKN55 and also consider organometallic compounds are missing. In the following, the DLPNO-MP2 implementation in the ORCA quantum chemistry software package^15,16^ is employed for B2PLYP as a representative DH functional resulting in the DLPNO-DH method DLPNO-B2PLYP. Its performance is evaluated against the conventional MP2-based B2PLYP functional for a selection of comprehensive benchmark sets for thermochemistry and molecule geometries.

## 2 Methods

### 2.1 DLPNO Accuracy Settings

DLPNO correlation methods are based on decomposition of the total correlation energy into contributions from electron pairs (pair correlation energies). Two principle approximations lead to linear scaling and high efficiency: (1) elimination of negligible electron pairs based on a highly efficient prescreening process that is based on the asymptotic expansion of the pair correlation energy. (2) Restriction of the virtual space to a local space spanned by projected atomic orbitals (PAOs) as well as compaction of this space through the natural orbital expansion for each electron pair separately. The accuracy of the first approximation is determined by a domain threshold *T*_CutDO_ and the second by the pair natural orbital threshold *T*_CutPNO_. The values of these two thresholds balance the accuracy of the approximation versus the computational cost. At default thresholds, typically more than 99.9% of the canonical correlation energy is recovered. For a detailed description of the DLPNO approximation in the context of MP2, we refer the interested reader to the literature.^6,11,12^ Similar to DLPNO-CCSD(T),^17^ default accuracy settings for DLPNO-MP2 are available in ORCA. These settings are employed for DLPNO-DH calculations as well, and the key truncation thresholds are shown in Table 1 (see Tables S1 and S2 in the SI for all truncation thresholds). Albeit *loosePNO* is not meant for accurate DLPNO-MP2 or DLPNO-DH calculations but rather for exploratory calculations, it was tested here because it is available in ORCA via a simple keyword and relevant in the context of PNO-space extrapolation. In contrast to DLPNO-CCSD(T), the accuracy thresholds are generally tighter. Additionally, compared to restricted references (RHF/RKS) tighter settings are required for unrestricted calculations (UHF/UKS). Therefore, in benchmark sets involving open-shell systems, the tighter thresholds were used for all systems including closed-shell systems.

**Table 1:** PNO Key Accuracy Settings for DLPNO-DHs

PNO-Settings	*T*_CutDO_ RKS/UKS	*T*_CutPNO_ RKS	*T*_CutPNO_ UKS
*loose*	2 · 10^–2^	10^–7^	10^–8^
*normal*	1 · 10^–2^	10^–8^	10^–9^
*tight*	5 · 10^–3^	10^–9^	10^–10^
*verytight*	2.5 · 10^–3^	10^–10^	10^–11^

For a fair assessment of the error introduced by the DLPNO-DH approximation, only errors with reference to the conventional MP2-based DH functional are discussed in the following. This means that no deviations from the original reference data of the investigated benchmark sets are discussed. The error is calculated according to eq 2:
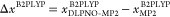
2

The resulting mean absolute deviation with regard to the conventional DH (MAD_C_) is calculated as
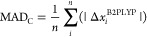
3

The MAD_C_ values are then employed to calculate the weighted mean absolute deviation (WTMAD-2_C_) according to
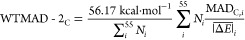
4

Here, 56.17 kcal·mol^–1^ is the average of the average absolute energies |Δ*E*|_*i*_ with the reference (B2PLYP) over all 55 sets of the GMTKN55, and *N*_*i*_ is the number of reactions with the MAD_C,*i*_ for the corresponding set *i* (See SI for details).

### 2.2 PNO-Space Extrapolation

The computational cost of any DLPNO-MP2 or DLPNO-DH calculation increases drastically upon tightening the *T*_CutPNO_ threshold. Accordingly, an extrapolation of the PNO space is desirable to obtain high accuracy at a reduced computational cost. The extrapolation to the complete PNO space (CPS) was successfully applied in the framework of local coupled cluster following eq 5.^18^ Here, *E*^*X*^ and *E*^*Y*^ are the energies (or properties) obtained with the respective *T*_CutPNO_ thresholds (e.g., *X* = 8 for *normalPNO* with *T*_CutPNO_ = 10^–8^ and *Y* = 9 for *tightPNO* with *T*_CutPNO_ = 10^–9^), *F* is an empirical scaling parameter, and *E*^*XY*^ is the extrapolated energy:
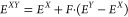
5

Furthermore, it has been shown that the CPS extrapolation reduces the size dependency of the DLPNO error in the context of DLPNO-CCSD(T).^19^ In a recent study by Kubas et al., a DLPNO-MP2 based extrapolation scheme for DLPNO-CCSD(T) has been proposed.^20^ Its good performance suggests that the DLPNO errors for MP2 and CCSD(T) are rather similar and that CPS extrapolation with a similar *F* parameter should be beneficial for DLPNO-MP2 and DLPNO-DHs as well. Therefore, in the following, the same *F* parameter (*F* = 1.5) that has been used for the DLPNO-CCSD(T) CPS extrapolation^18^ was assessed for CPS extrapolation in DLPNO-B2PLYP. In this work, *F* = 1.5 proved suitable also for DLPNO-DH calculations supporting the findings of Kubas et al. In the following, the nomenclature for CPS extrapolation will be CPS(*X*→*Y*) with abbreviations *l* for *loosePNO*, *n* for *normalPNO*, *t* for *tightPNO*, and *vt* for *verytightPNO*.

### 2.3 Computational Details

All calculations were performed with ORCA version 5.0.4^15,16^ employing the B2PLYP DH functional^5^ either with the DLPNO approximation (DLPNO-B2PLYP) or with the conventional resolution of the identity (RI)-B2PLYP method^21,22^ in combination with the def2-TZVPP triple-ζ basis^23,24^ with the corresponding def2-TZVPP/C auxiliary basis. As an integration grid for the DFT calculations, the large *DEFGRID3* was employed, and for the SCF, *TightSCF* settings were selected. Additionally, the Split-RI-J^25^ and RIJCOSX^26^ approximations were used to speed up the calculations. The frozen core approximation with default settings was used throughout.

## 3 Results and Discussion

### 3.1 Thermochemistry

**Figure 1:**
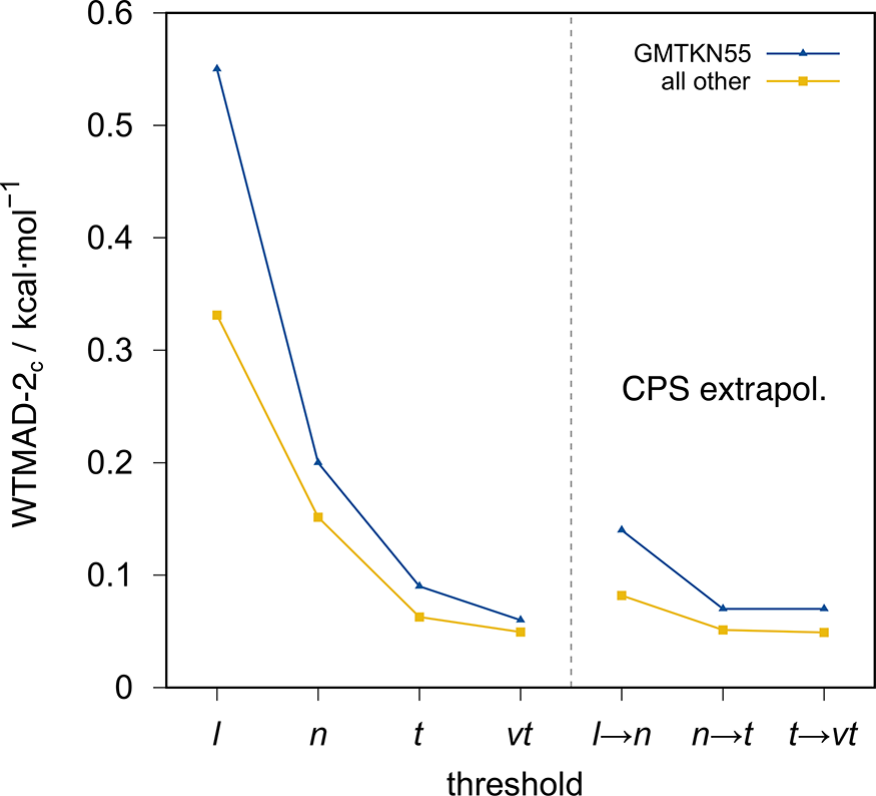
WTMAD-2_C_ with reference to conventional B2PLYP in kcal·mol^–1^ for the GMTKN55 benchmark set collection and all other thermochemistry benchmark sets assessed (cf. Table 3).

The general main-group thermochemistry, kinetics, and noncovalent interactions (NCIs) database (GMTKN55)^60^ was employed to investigate the influence of the DLPNO-DH approximation on general main-group thermochemistry. The WTMAD-2_C_ for different PNO thresholds on the whole GMTKN55 and on the respective subsets with reference to conventional B2PLYP is shown in Table 2. The WTMAD-2_C_ for the whole GMTKN55 is also depicted in Figure 1.

**Table 2:** WTMAD-2_C_ of DLPNO-B2PLYP on the GMTKN55 Database in kcal·mol^–1^

Set	#	*loose*	*normal*	*tight*	*verytight*	CPS(*l*→*n*)	CPS(*n*→*t*)	CPS(*t*→*vt*)
basic	473	0.06	0.03	0.02	0.02	0.03	0.02	0.02
reactions	243	0.47	0.15	0.07	0.05	0.09	0.05	0.04
barriers	194	0.10	0.04	0.04	0.05	0.05	0.05	0.05
inter NCIs	304	1.28	0.47	0.21	0.13	0.25	0.15	0.14
intra. NCIs	291	0.95	0.32	0.15	0.08	0.30	0.11	0.09
GMTKN55	1505	0.55	0.20	0.09	0.06	0.14	0.07	0.07

For the whole GMTKN55 database, the largest WTMAD-2_C_ decrease is observed from *loosePNO* (0.55 kcal·mol^–1^) to *normalPNO* (0.20 kcal·mol^–1^) settings, and smaller further reductions are obtained with *tightPNO* (0.09 kcal·mol^–1^) and *verytightPNO* (0.06 kcal·mol^–1^) settings. In none of the subsets are the WTMAD-2_C_ values above 1 kcal·mol^–1^ except for the intermolecular NCIs when *loosePNO* is employed. For the basic properties subset, *loosePNO* yields only a tiny WTMAD-2_C_ (0.06 kcal·mol^–1^). Here, only minor improvements in WTMAD-2_C_ can be obtained by going up to *verytightPNO* (0.02 kcal·mol^–1^). This is because the basic property subset mostly contains small molecules. For the reactions subset, the WTMAD-2_C_ with *loosePNO* is larger (0.47 kcal·mol^–1^) and still present with *normalPNO* (0.15 kcal·mol^–1^), but it becomes negligible with *tightPNO* (0.07 kcal·mol^–1^) and *verytightPNO* (0.05 kcal·mol^–1^) settings. Small errors are also observed for barriers with the WTMAD-2_C_ for *loosePNO* already being tiny (0.10 kcal·mol^–1^) with small improvements with *normalPNO* (0.04 kcal·mol^–1^) but no further improvements with even tighter settings. Larger deviations are observed for the inter- and intramolecular NCI subsets where *loosePNO* yields WTMAD-2_C_ values around 1 kcal·mol^–1^. These WTMAD-2_C_ values are reduced to a third by employing *normalPNO* (0.47 kcal·mol^–1^ and 0.32 kcal·mol^–1^) and further halved by using *tightPNO* (0.21 kcal·mol^–1^ and 0.15 kcal·mol^–1^) and *verytightPNO* (0.13 kcal·mol^–1^ and 0.08 kcal·mol^–1^) settings. The CPS extrapolation generally reduces the WTMAD-2_C_ for CPS(*l*→*n*) and CPS(*n*→*t*), but no improvement is observed for CPS(*t*→*vt*). Since the errors with *tightPNO* are almost converged with regard to the PNO thresholds, no further improvement is obtained by CPS(*t*→*vt*) extrapolation in this case. The improvement from *normalPNO* to CPS(*l*→*n*) is larger (from 0.20 to 0.14 kcal·mol^–1^) than that from *tightPNO* to CPS(*n*→*t*) (from 0.09 to 0.07 kcal·mol^–1^). For the GMTKN55, *tightPNO* and tighter settings and CPS(*n*→*t*) and higher can be considered as converged, because WTMAD-2_C_ values smaller than 0.1 kcal·mol^–1^ are obtained. Such errors are negligible for practical applications, in comparison to the overall DH errors.

In addition to the GMTKN55 database, several benchmark sets were considered. The results are shown in Table 3 and an overall weighted MAD_C_ in Figure 1. These include sets for NCIs of large systems (L7,^38,39^ S30L,^40^ and HS13L^41^), ion-π interactions (IONPI19^27^), halogen bonds (X40×10^30^), hydrogen bonds (HB300SPX^34^), chalcogen bonds (CHAL336^32^), frustrated Lewis pairs (LP14), conformational energies of alkanes (ACONF-L^33^), and repulsive NCIs (R160×6^28^). For barrier heights and reaction energies, the revBH9^35,36^ set is included. Also included are sets containing transition metal complexes for closed-shell reaction energies (MOR41^42^ and WCCR10^44,45^), open-shell reaction energies (ROST61^43^), conformational energies (TMCONF16^46^), barrier heights (MOBH35^51,52^ and TMBH^47−50^), and ionization energies (TMIP^54^).

**Table 3:** Benchmark Sets Included in the Assessment of DLPNO-B2PLYP with the Respective MAD_C_ in kcal·mol^–1^^a^

Set	#	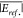	*l*	*n*	*t*	*vt*	CPS(*l*→*n*)	CPS(*n*→*t*)	CPS(*t*→*vt*)
IONPI19^27^	19	20.87	0.23	0.10	0.04	0.02	0.04	0.02	0.01
R160×6^28,29^	960	2.04	0.02	0.01	0.00	0.00	0.01	0.00	0.00
X40×10^30^	400	2.73	0.05	0.02	0.01	0.00	0.01	0.01	0.00
CHAL336^32^	336	14.09	0.12	0.05	0.02	0.01	0.02	0.01	0.01
ACONF-L^33^	50	4.62	0.21	0.08	0.04	0.02	0.03	0.01	0.01
HB300SPX^34^	3000	3.18	0.04	0.02	0.01	0.01	0.01	0.01	0.01
revBH9_BH_^35,36^	898	20.37	0.31	0.12	0.05	0.04	0.05	0.02	0.04
revBH9_RE_^35,36^	449	11.08	0.19	0.08	0.03	0.02	0.04	0.02	0.03
LP14^37^	14	23.33	1.04	0.48	0.23	0.10	0.21	0.11	0.04
L7^38,39^	7	16.27	1.34	0.60	0.24	0.08	0.23	0.08	0.02
S30L^40^	30	37.51	2.52	1.22	0.60	0.26	0.57	0.28	0.10
HS13L^*a*^^41^	13	45.82	2.29	1.14	0.57	0.28	0.57	0.29	0.13
MOR41^42^	41	31.20	0.60	0.26	0.11	0.05	0.10	0.04	0.02
ROST61^43^	61	42.78	0.40	0.18	0.09	0.05	0.08	0.05	0.03
WCCR10^44,45^	10	48.72	1.12	0.51	0.22	0.09	0.21	0.10	0.04
TMCONF16^46^	16	3.15	0.04	0.02	0.01	0.00	0.03	0.01	0.00
TMBH^47−50^	40	14.47	0.14	0.05	0.02	0.01	0.02	0.01	0.01
MOBH35^51−53^	70	20.89	0.25	0.09	0.04	0.02	0.04	0.02	0.01
TMIP^54^	11	95.62	0.58	0.28	0.13	0.06	0.14	0.06	0.03

a PNO settings are abbreviated (*l*, *n*, *t*, *vt*).  is the original mean absolute reference energy of the respective benchmark sets.

The largest errors are obtained for the NCI sets containing large systems. For the S30L, *loosePNO* yields an MAD_C_ of 2.52 kcal·mol^–1^ that is larger than the MAD_C_s of the best performing DFT methods for this set (around 2 kcal·mol^–1^). Tightening the PNO settings successively halves the MAD_C_ for this set from *normalPNO* (1.22 kcal·mol^–1^) to *tightPNO* (0.60 kcal·mol^–1^) and to *verytightPNO* (0.26 kcal·mol^–1^). Here, CPS(*t*→*vt*) yields a basically converged MAD_C_ of 0.10 kcal·mol^–1^, but due to the many π–π interactions in the S30L (as for the HS13L, L7, and LP14) the application of DH functionals to this set is questionable in the first place. Similar behavior as for the S30L is observed for the HS13L. Less prone but still severe are the MAD_C_s of the L7 and the LP14 sets where the MAD_C_s (as the average interaction energies) are basically halved compared to the S30L and HS13L. Much smaller are the MAD_C_s for the IONPI19, the revBH9, and the ACONF-L set where MAD_C_s of 0.1 kcal·mol^–1^ are already reached with *normalPNO* except for the barriers of the revBH9 by 0.02 kcal·mol^–1^. The MAD_C_s of the CHAL336, X40×10, HB300SPX, and R160×6 sets are already small with *loosePNO* and become vanishingly small with tighter settings. This may again be attributed to the relatively small system size of the molecules in these sets. For the transition metal containing sets, the WCCR10 shows the largest MAD_C_s (1.12 kcal·mol^–1^ with *loosePNO*) followed by the MOR41, the TMIP, and the ROST61 (between 0.4–0.6 kcal·mol^–1^ with *loosePNO*). Smaller errors are observed for the MOBH35 and the TMBH, where all settings tighter than *loosePNO* yield MAD_C_s smaller than 0.1 kcal·mol^–1^. Surprising are the vanishing MAD_C_s for the TMCONF16 set. In conclusion, the errors for the organometallic sets are larger than for typical organic reactions, but with *tightPNO* settings or CPS(*l*→*n*), the MAD_C_s are around 0.1 kcal·mol^–1^ (with one exception). This error is negligible compared to the errors of the corresponding DH.

### 3.2 MP2 and HFX Contribution

**Figure 2:**
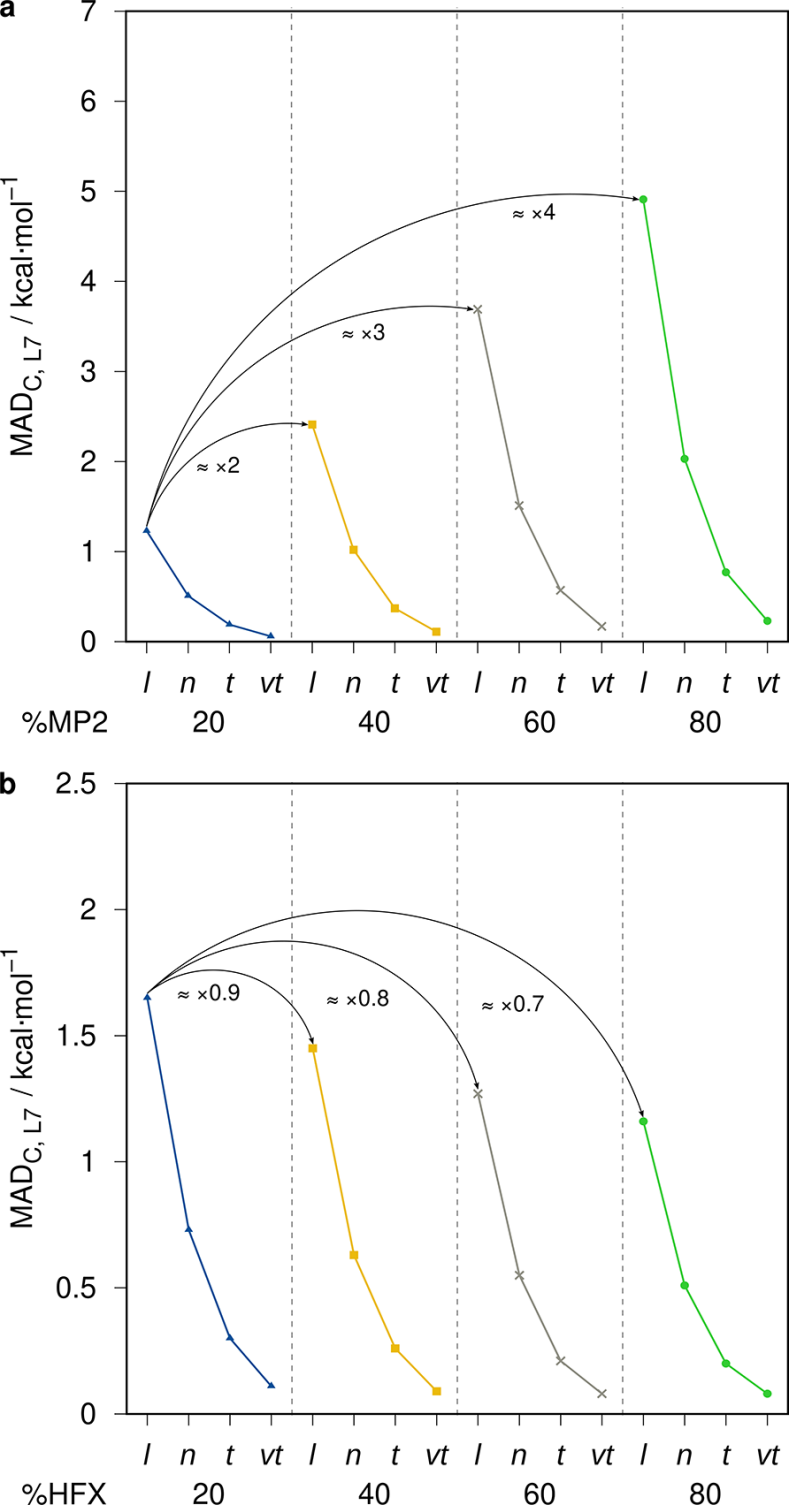
(a) MAD_C_s for the L7 benchmark set for B2PLYP variants with varying amounts of MP2 correlation. (b) MAD_C_s for the B2PLYP variant with varying amounts of HFX. *l* = *loosePNO*, *n* = *normalPNO*, *t* = *tightPNO*, and *vt* = *verytightPNO*.

As DH functionals typically include different amounts of MP2 correlation in their energy expression (cf. eq 1), the estimated error introduced by the DLPNO-DH approximation can vary as well. Nevertheless, the introduced error behaves linearly with the amount of MP2 correlation, which is demonstrated for DLPNO-B2PLYP variants with varying amounts of MP2 of 20, 40, 60, and 80% on the L7 set (Figure 2a). Accordingly, tripling the amount of MP2 correlation triples the DLPNO-DH error with respect to conventional B2PLYP. Nevertheless, most robust and well-behaved DHs employ values of around 30% MP2 correlation, allowing for a reasonable error estimate based on the results for B2PLYP (27% MP2). In addition to different amounts of MP2, different amounts of HFX are employed as well in DH functionals, which can influence the DLPNO error. This is demonstrated for DLPNO-B2PLYP variants with varying amounts of HFX of 20, 40, 60, and 80% on the L7 set (Figure 2b). With increasing amounts of HFX, the self-interaction error in the DH functionals is reduced, which results in a reduced level of electron delocalization and a slightly reduced DLPNO error. The most robust and well-behaved DHs employ between 50–80% HFX and can therefore benefit from a slightly reduced DLPNO error compared to DHs with less HFX.

### 3.3 Size Dependence of the Correlation Energy Error

**Figure 3:**
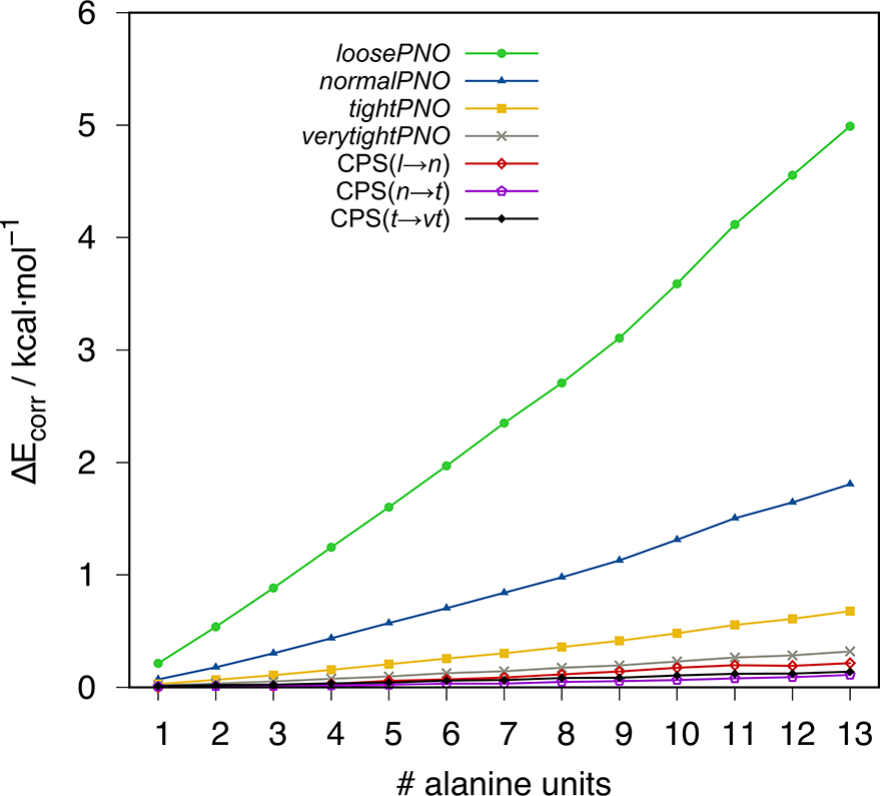
Error in MP2 correlation energy with reference to conventional B2PLYP in kcal·mol^–1^ for polyalanines.

In line with previous findings on PNO errors in DLPNO-CCSD(T) approaches, the correlation energy error with respect to conventional B2PLYP behaves almost linearly with the size of the system. This is demonstrated for a polyalanine chain (Figure 3) where a clear decrease in the size dependence upon tightening the PNO thresholds is observed. Further, even a CPS(*l*→*n*) PNO space extrapolation can eliminate most of the size-dependent correlation energy error for this case.

### 3.4 Geometry Optimizations

**Figure 4:**
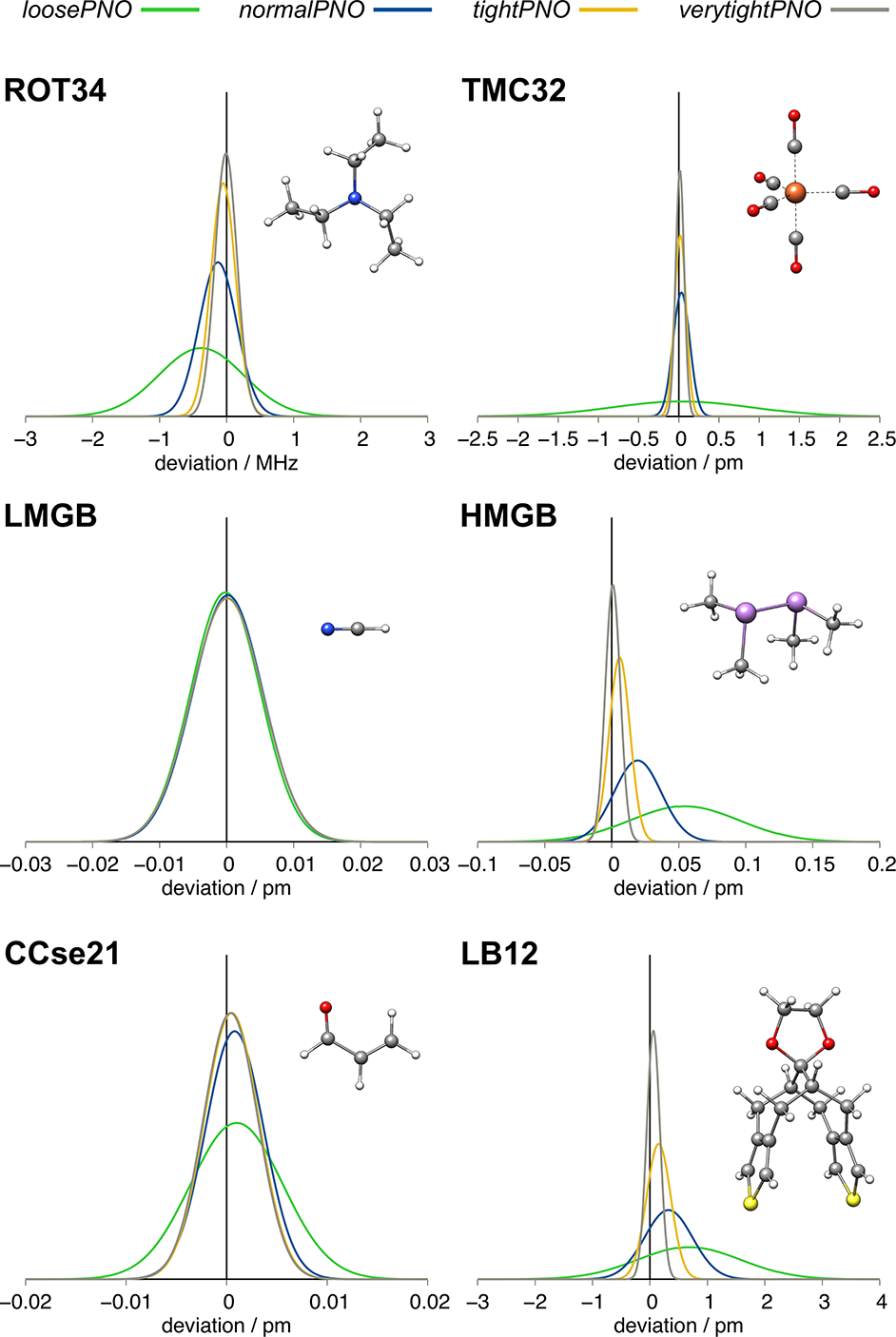
Gaussian error distributions for selected bond length benchmark sets with reference to conventional B2PLYP results. Negative mean deviations indicate overall too short bond lengths compared with the canonical result.

**Figure 5:**
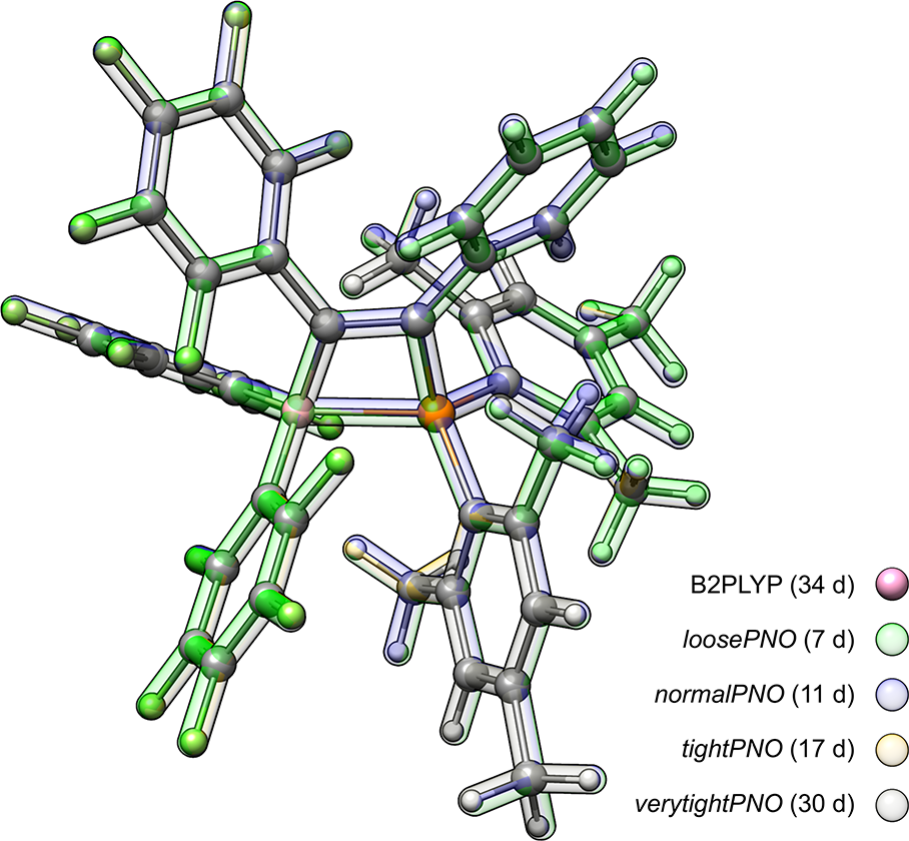
Structure overlay of FLP (88 atoms) from the LB12 benchmark set optimized at various PNO threshold settings. All optimizations were performed on 4 CPUs using an Intel Xeon CPU E3-1270 v5 @ 3.60 GHz machine.

As even energy calculations on a high theoretical level such as DH-DFT are computationally demanding, geometry optimizations requiring many energy and gradient evaluations are typically unfeasible. Nevertheless, highly accurate geometry optimizations are desirable for critical cases and specifically benchmarking more approximate methods such as semiempirical quantum mechanics (SQM) or force-fields (FF). By employing DLPNO-DH, respective DH functionals become feasible again for geometry optimizations of medium-sized to large molecules. To estimate the influence of the DLPNO-DH threshold settings, we performed DLPNO-B2PLYP geometry optimizations for various established geometry optimization benchmark sets. The resulting geometries were compared to the conventional MP2-based B2PLYP results (Figure 4 and Table 4). The following geometric features were investigated: Rotational constants for small to medium-sized organic molecules were compared (ROT34^57^). Bond lengths were compared for 3d transition metal complexes (TMC32^59^) and light (LMGB35^58^) and heavy (HMGB11^58^) main-group compounds as well as a mixed set containing unusually long bonds (LB12^58^). Additionally, for the CCe21 set,^55,56^ containing semiexperimental structures of organic molecules, bond distances and angles were compared. No effect of the accuracy settings is observed for the LMGB35, and the conventional B2PLYP bond lengths are almost obtained with a vanishing MAD_C_ of 0.003 pm, because the molecules in this test set are very small. Similar errors are observed for the CCse21 and the HMGB11 sets. Although here small differences between the PNO settings are observed, larger errors that are still below 1 pm are found for TMC32 and the LB12 sets with *loosePNO*. For the TMC32 set, *normalPNO* is already sufficient, while for the LB12 set, errors below 0.1 pm are only obtained with *verytightPNO*. For the ROT34, small MAD_C_s are observed with *loosePNO* (0.526 MHz) and *normalPNO* (0.224 MHz) and basically vanish with *tightPNO* (0.051 MHz). In general, the introduced errors of the DLPNO approximation are very small compared to those of the B2PLYP result. In all cases, MAD_C_s below 1 pm, 1 MHz, or 1 [°] were obtained. The very small differences between the structures obtained by using varying PNO thresholds and conventional B2PLYP can also be seen for the large frustrated Lewis-pair (FLP) system of the LB12 benchmark set. An overlay of all optimized structures shows no significant difference in the optimized structures (Figure 5), underlining the value of using less tight PNO thresholds for geometry optimizations. Overall, the errors introduced by the DLPNO-DH approximation are generally much less pronounced for geometrical features. This renders the efficient *normalPNO* settings already suitable for DLPNO-DH geometry optimizations of large systems.

**Table 4:** Geometry Benchmark Sets Included in the Assessment of DLPNO-B2PLYP^a^

			*loose*		*normal*		*tight*		*verytight*	
Set	#		MAD_C_	MD_C_	MAD_C_	MD_C_	MAD_C_	MD_C_	MAD_C_	MD_C_
CCse21_bonds_^55,56^	68	122.33	0.003	0.001	0.002	0.001	0.002	0.000	0.002	0.000
CCse21_angles_^55,56^ [°]	42	116.03	0.004	0.000	0.004	0.000	0.004	0.000	0.004	0.000
ROT34^b^^57^ [MHz]	34	1411.72	0.526	–0.379	0.224	–0.129	0.051	–0.050	0.013	–0.009
HMGB11^58^	11	243.40	0.055	0.055	0.019	0.019	0.006	0.006	0.004	0.001
LMGB35^b^^58^	26	114.01	0.003	0.000	0.003	0.000	0.003	0.000	0.003	0.000
LB12^58^	12	299.26	0.701	0.689	0.339	0.323	0.164	0.152	0.083	0.059
TMC32^b^^,^^c^^59^	46	189.47	0.298	0.047	0.070	0.033	0.035	0.020	0.024	0.015

a Mean absolute deviations (MAD_C_) and mean deviations (MD_C_) in pm, °, or MHz. All deviations are given relative to the conventional MP2-based DH functional.  is the original mean absolute reference structural property of the respective benchmark sets.

b Open-shell systems were excluded as no gradient is yet available for them.

c Fe(CO)_2_(NO)_2_ was excluded due to convergence problems with B2PLYP.

### 3.5 Timing Comparisons

**Figure 6:**
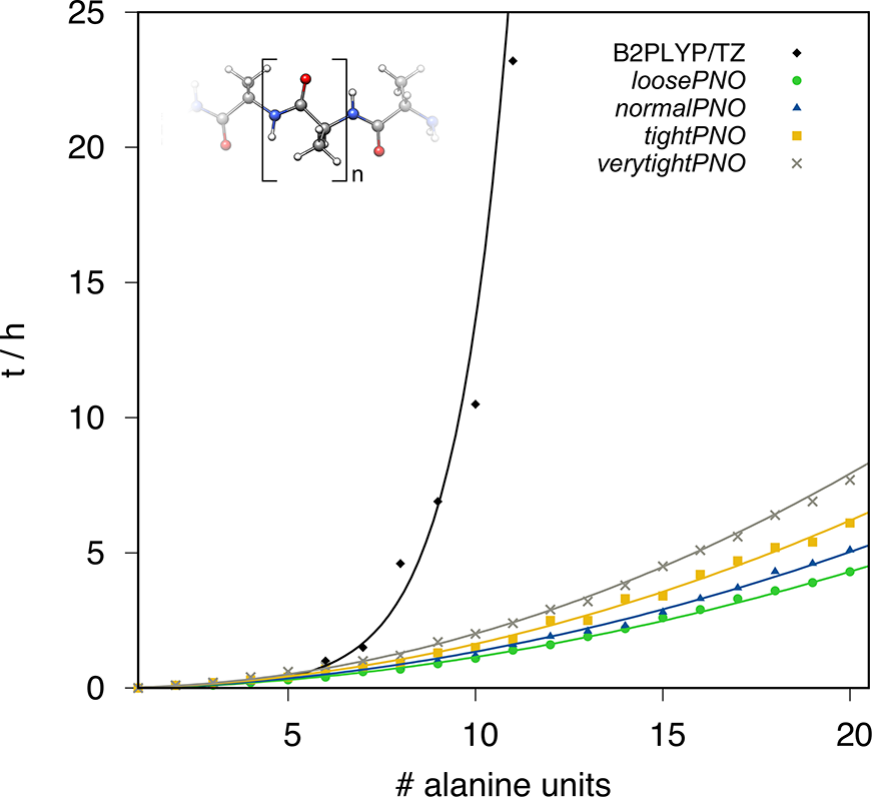
Computation wall-times in h for energy and gradient evaluation of polyalanine chains with up to 20 alanine units (203 atoms) for conventional B2PLYP/def2-TZVP and DLPNO-B2PLYP/def2-TZVP with different PNO thresholds. All calculations were performed on 14 CPUs using an Intel Xeon CPU E5-2660 v4 @ 2.00 GHz machine.

**Figure 7:**
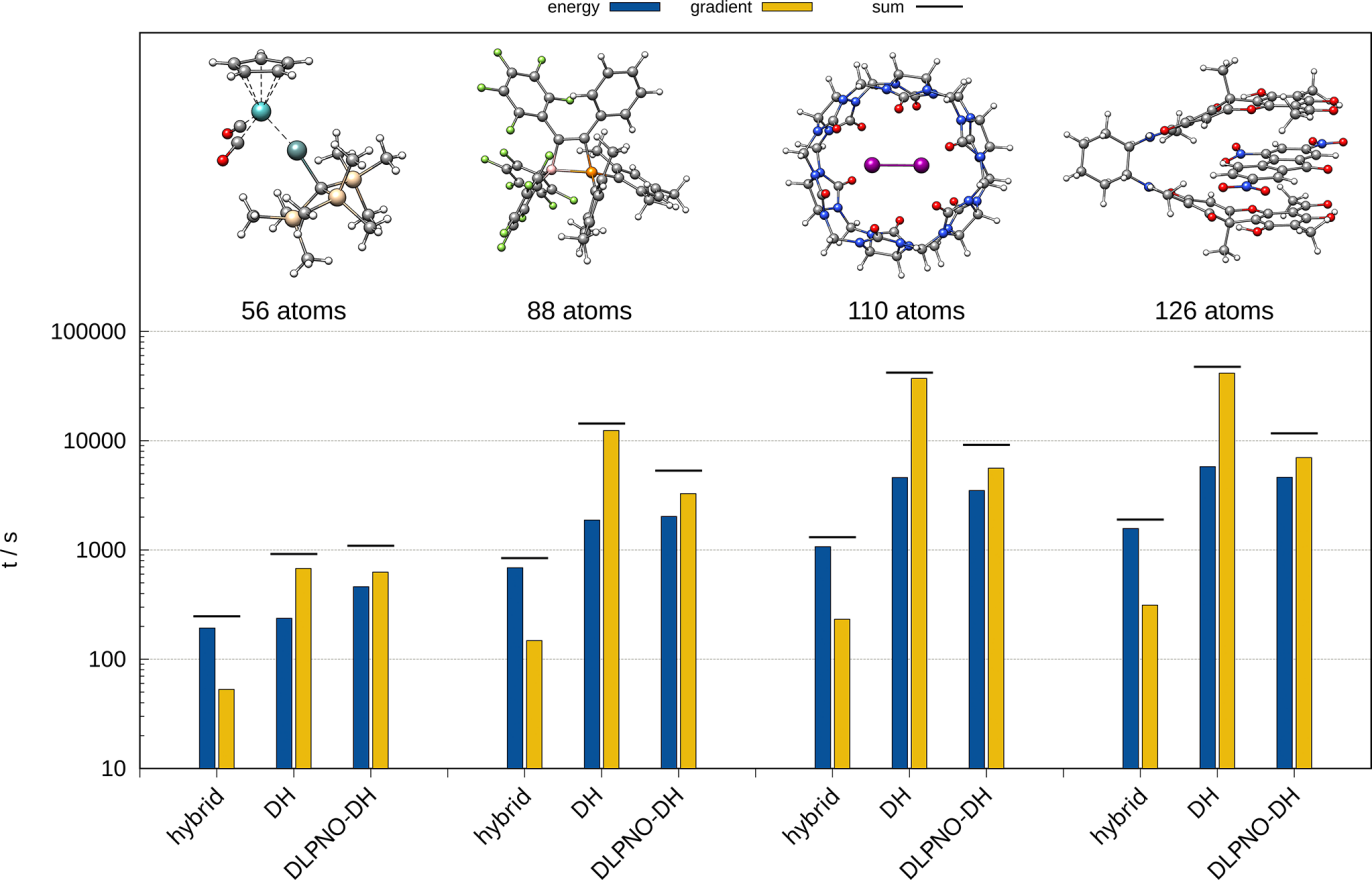
Computation times in s for energy and gradient evaluation of selected molecules in the range of 56 to 126 atoms. Hybrid = BLYP with 53% HFX; DH = B2PLYP; and DLPNO-DH = DLPNO-B2PLYP with *normalPNO* thresholds. The def2-TZVP(-f) basis was used throughout. All calculations were performed on 14 CPUs using an Intel Xeon CPU E5-2660 v4 @ 2.00 GHz machine. Note the logarithmic scale.

The computational demand of energy and gradient evaluations typically determines the feasibility of geometry optimization. Therefore, the computational wall-time reduction of a subsequent energy and gradient calculation is assessed for various PNO thresholds for polyalanine with varying chain lengths (Figure 6). At a crossing point of about five alanine units (53 atoms), the DLPNO-DH approximation begins to drastically reduce the computation time of the combined energy and gradient compared to the conventional DH. The steep scaling of the latter causes a drastic increase in computation time, while the DLPNO-DH approach yields a flat, almost linear, scaling with the size of the system. A comparison of the energy and gradient computation time contributions for the parental hybrid functional and the DH variants is depicted in Figure 7. The scaling of the conventional and the DLPNO-DH with respect to the system size (number of basis functions) for these selected molecules is shown in Figure S1 in SI. In line with the results shown for the polyalanine chain, the gradient evaluation profits significantly from the DLPNO-DH approximation, even for medium sized molecules such as the depicted molybdosilylidine complex with 56 atoms. Nevertheless, for these molecule sizes, the overhead of the DLPNO space construction causes more costly energy evaluations compared to the conventional DH, thus resulting in a higher overall computation time. With increasing size, the energy computation using the DLPNO approximation becomes increasingly faster, and the benefit for the gradient evaluation is even more drastic underlining the value of DLPNO-DH calculations for molecules with more than 100 atoms.

### 3.6 General Recommendations

Finally, as a good compromise between computational cost and accuracy, we recommend employing *normalPNO* for the gradient calculation of medium-sized organic compounds (up to 100 atoms) and conventional DHs for the energy evaluation, as the latter does not profit from the DLPNO approximation speedup. In the regime of 100 atoms and larger, DLPNO-DHs yield an increasing speedup and may be employed with *normalPNO* for most systems. In this context, the DLPNO approximation starts to generally enable DH calculations that would be unfeasible for such large systems due to computation time and memory issues. In terms of PNO-space extrapolation, we recommend CPS(*l*→*n*) for reactions and CPS(*n*→*t*) for noncovalent interactions.

## 4 Conclusion

In this work, the application of the DLPNO-MP2 approximation to the DH-DFT framework was assessed. The performance of different PNO thresholds as well as PNO space extrapolations was tested for the prominent B2PLYP functional on various benchmark sets for the thermochemistry of main-group molecules and transition metal complexes. It was demonstrated that *tightPNO* settings yield reliably small deviations from conventional B2PLYP at a drastically reduced computational cost for large systems (WTMAD-2_C_^all^ = 0.06 kcal·mol^–1^, WTMAD-2_C_^GMTKN55^ = 0.09 kcal·mol^–1^).

In general, we expect that the observed DLPNO error is transferable to other DH functionals as the error behaves linearly with the amount of the MP2 correlation and HFX admixture. The errors for geometry optimizations were found to be even smaller and in many cases negligibly small, even at moderately tight PNO thresholds. *normalPNO* yields already satisfactory agreement with geometries optimized with conventional B2PLYP. The CPS extrapolation scheme introduced in the DLPNO-CCSD(T) framework was successfully applied to DLPNO-DH calculations, with CPS(*n*→*t*) typically yielding accurate results with very small residual errors compared to the conventional DHs (WTMAD-2_C_^all^ = 0.05 kcal·mol^–1^, WTMAD-2_C_^GMTKN55^ = 0.07 kcal·mol^–1^). The CPS parameter *F* = 1.5 was also found to be suitable in the DLPNO-DH-DFT framework.

The performance of DLPNO-DH-DFT can potentially also benefit from employing the so-called tightened semicore settings as proposed by Altun et al.^61^ for transition metal complexes or modified PNO settings as proposed by Werner and Hansen.^62^

Overall, it is demonstrated that DLPNO-DH-DFT represents a valuable alternative to conventional DH-DFT for large systems where the unfavorable *N*^5^ scaling of MP2 prevents its application. DLPNO-DH-DFT may be applied to enable highly accurate geometry optimizations and energy calculations of large molecules that are not feasible with conventional DH functionals. Further, the technical implementation of the underlying DLPNO-MP2 is much easier compared to its DLPNO-CCSD(T) counterpart, increasing its potential availability in common quantum chemistry programs.

## Supporting Information Available

Detailed DLPNO thresholds, statistical measures, and additional statistics for all benchmark sets (PDF)
