# Supplementary material for: Assessment of DLPNO-MP2 Approximations in Double-Hybrid DFT
Source: J Chem Theory Comput. 2023 Oct 20;19(21):7695–703. doi: 10.1021/acs.jctc.3c00896 (PMC10653103; doi:10.1021/acs.jctc.3c00896)
Supplement: Supplementary file 1 — ct3c00896_si_001.pdf [file ct3c00896_si_001.pdf]

Supporting Information: Assessment of DLPNO-MP2 Approximations in Double-Hybrid DFT

Hagen Neugebauer,[†] Peter Pinski,[‡] Stefan Grimme,[†] Frank Neese,[*,¶] and Markus Bursch[*,¶]

† *Mulliken Center for Theoretical Chemistry, Clausius Institute for Physical and Theoretical Chemistry, University of Bonn, Beringstr. 4, D-53115 Bonn, Germany*

‡*HQS Quantum Simulations GmbH, Rintheimer Straße 23, D-76131 Karlsruhe, Germany*

¶*Max-Planck-Institut für Kohlenforschung, Kaiser-Wilhelm-Platz 1, D-45470 Mülheim an der Ruhr, Germany*

E-mail: neese@kofo.mpg.de; bursch@kofo.mpg.de

1 DLPNO-DH accuracy settings

Table S1: All truncation thresholds for the different accuracy settings of RKS DLPNO-DHs. Thresholds that occur only once in a row apply to the whole row.

Threshold	*loosePNO*	*normalPNO*	*tightPNO*	*verytightPNO*
T_{CutPNO}	1×10^{-7}	1×10^{-8}	1×10^{-9}	1×10^{-10}
T_{CutDO}	2×10^{-2}	1×10^{-2}	5×10^{-3}	2.5×10^{-3}
T_{CutMKN}		1×10^{-3}		
F_{Cut}		1×10^{-5}		
T_{CutPre}		1×10^{-6}		
T_{CutDOIJ}		1×10^{-5}		
T_{CutDOPre}		3×10^{-2}		
T_{CutC}		1×10^{-3}		

Table S2: All truncation thresholds for the different accuracy settings of UKS DLPNO-DHs. Thresholds that occur only once in a row apply to the whole row.

Threshold	*loosePNO*	*normalPNO*	*tightPNO*	*verytightPNO*
T_{CutPNO}	1×10^{-8}	1×10^{-9}	1×10^{-10}	1×10^{-11}
T_{CutDO}	2×10^{-2}	1×10^{-2}	5×10^{-3}	2.5×10^{-3}
T_{CutMKN}		1×10^{-3}		
F_{Cut}		1×10^{-5}		
T_{CutPre}		1×10^{-6}		
T_{CutDOIJ}		1×10^{-5}		
T_{CutDOPre}		3×10^{-2}		
T_{CutC}		1×10^{-3}		

2 Statistical errors measures

$$\Delta x^{B2PLYP} = x^{B2PLYP}_{DLPNO-MP2} - x^{B2PLYP}_{MP2}. \tag{1}$$

$$\mathrm{MD_C} = \frac{1}{N}\sum_{i}^{N} \Delta x_i^{B2PLYP} \tag{2}$$

$$\mathrm{MAD_C} = \frac{1}{n}\sum_{i}^{n}(|\Delta x_i^{B2PLYP}|). \tag{3}$$

$$\mathrm{SD_C} = \sqrt{\frac{\sum_i^N \mid \Delta x_i^{B2PLYP} - \mathrm{MD_C} \mid^2}{N-1}} \tag{4}$$

$$\mathrm{AMAX_C} = MAX(|\Delta x_i^{B2PLYP}|) \tag{5}$$

3 Scaling for selected molecules

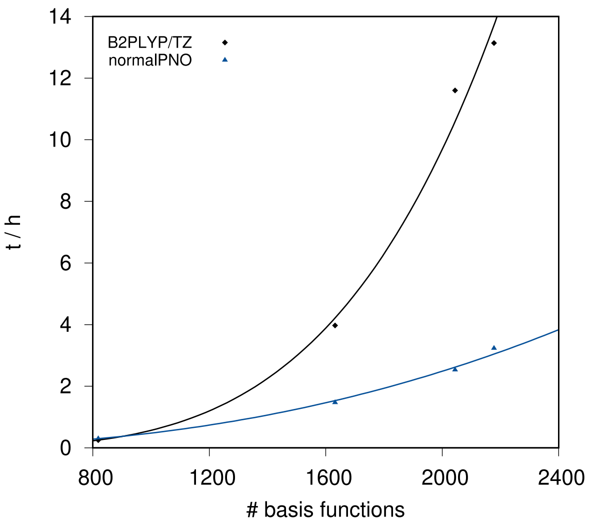

Figure S1: Computation wall-times in h for energy and gradient evaluation with conventional and DLPNO-B2PLYP/def2-TZVP(-f) (with *normalPNO* thresholds) for selected molecules in the range of 56 to 126 atoms (shown in Figure 7 of the manuscript) with respect to the number of basis functions.

4 GMTKN55

The WTMAD-2$_\mathrm{C}$ values were calculated according to

$$\text{WTMAD-2}_\mathrm{C} = \frac{56.17\ \text{kcal}\cdot\text{mol}^{-1}}{\sum_i^{55} N_i} \sum_i^{55} N_i \frac{MAD_{C,i}}{\overline{|\Delta E|}_i}. \tag{6}$$

The WTMAD-2$_\mathrm{C}^\mathrm{subset}$ values for the subsets were calculated according to:

$$\text{WTMAD-2}_\mathrm{C}^\mathrm{subset} = \frac{56.17\ \text{kcal}\cdot\text{mol}^{-1}}{\sum_i^{\text{subset}} N_i} \sum_i^{\text{subset}} N_i \frac{\text{MAD}_{\text{C},i}}{\overline{|\Delta E|}_i}. \tag{7}$$

The corresponding MAD$_\mathrm{C}$, N_i and $\overline{|\Delta E|}_i$ values are given in Table S3 and S4.

Table S3: MAD_C values of the GMTKN55 with different PNO settings (Part 1) in kcal·mol^{-1}.

Set	N_i	$\overline{\|\Delta E\|}_i$	*loose*	*normal*	*tight*	*vtight*	$l \rightarrow n$	$n \rightarrow t$	$t \rightarrow vt$
W4-11	140	303.45	0.09	0.03	0.03	0.04	0.03	0.03	0.04
G21EA	25	26.11	0.03	0.03	0.03	0.03	0.03	0.03	0.03
G21IP	36	256.02	0.02	0.02	0.02	0.02	0.02	0.02	0.02
DIPCS10	10	649.98	0.02	0.01	0.00	0.00	0.01	0.01	0.00
PA26	26	190.43	0.01	0.01	0.01	0.00	0.01	0.01	0.00
SIE4x4	16	43.77	0.05	0.04	0.03	0.03	0.04	0.03	0.03
ALKBDE10	10	96.89	0.08	0.06	0.05	0.04	0.06	0.05	0.03
YBDE18	18	45.67	0.08	0.03	0.01	0.01	0.01	0.01	0.01
AL2X6	6	30.26	0.11	0.03	0.01	0.01	0.01	0.01	0.01
HEAVYSB11	11	53.21	0.14	0.05	0.02	0.02	0.03	0.01	0.02
NBPRC	12	25.23	0.05	0.02	0.01	0.01	0.02	0.02	0.02
ALK8	8	58.74	0.10	0.02	0.01	0.00	0.02	0.00	0.00
RC21	21	34.39	0.04	0.02	0.02	0.02	0.02	0.02	0.02
G2RC	25	50.30	0.04	0.01	0.01	0.01	0.01	0.01	0.01
BH76RC	30	21.59	0.02	0.02	0.02	0.02	0.03	0.02	0.02
FH51	51	29.87	0.05	0.02	0.01	0.01	0.01	0.01	0.01
TAUT15	15	3.54	0.01	0.01	0.00	0.00	0.01	0.01	0.00
DC13	13	50.80	0.27	0.14	0.09	0.06	0.10	0.06	0.05
MB16-43	43	433.15	0.26	0.20	0.34	0.39	0.34	0.42	0.41
DARC	14	24.29	0.32	0.07	0.02	0.01	0.05	0.01	0.01
RSE43	43	8.19	0.01	0.01	0.01	0.01	0.01	0.01	0.01
BSR36	36	11.00	0.34	0.10	0.04	0.01	0.03	0.01	0.01
CDIE20	20	4.43	0.02	0.01	0.01	0.01	0.01	0.01	0.01
ISO34	34	14.67	0.04	0.01	0.01	0.01	0.01	0.01	0.01
ISOL24	24	19.55	0.20	0.08	0.03	0.01	0.04	0.01	0.01
C60ISO	9	91.86	1.64	0.59	0.26	0.22	0.08	0.10	0.20
PArel	20	4.69	0.01	0.01	0.00	0.00	0.01	0.01	0.00

Table S4: MAD_C values of the GMTKN55 with different PNO settings (Part 2) in kcal·mol^{-1}.

Set	N_i	$\overline{\lvert\Delta E\rvert}_i$	*loose*	*normal*	*tight*	*vtight*	$l \to n$	$n \to t$	$t \to vt$
BH76	76	17.21	0.02	0.02	0.02	0.03	0.02	0.03	0.03
BHPERI	26	21.24	0.10	0.02	0.01	0.01	0.02	0.01	0.01
BHDIV10	10	44.74	0.04	0.01	0.01	0.00	0.01	0.01	0.01
INV24	24	31.08	0.06	0.02	0.01	0.01	0.03	0.01	0.01
BHROT27	27	6.49	0.01	0.00	0.00	0.00	0.01	0.01	0.00
PX13	13	30.36	0.03	0.01	0.01	0.01	0.01	0.01	0.01
WCPT18	18	33.46	0.05	0.02	0.01	0.01	0.01	0.01	0.01
RG18	18	0.21	0.01	0.00	0.00	0.00	0.00	0.00	0.00
ADIM6	6	0.50	0.17	0.06	0.03	0.01	0.02	0.01	0.01
S22	22	5.66	0.12	0.03	0.01	0.01	0.02	0.01	0.01
S66	66	4.00	0.09	0.03	0.01	0.01	0.01	0.01	0.01
HEAVY28	28	0.62	0.02	0.01	0.01	0.00	0.01	0.01	0.01
WATER27	27	88.00	0.19	0.08	0.05	0.03	0.03	0.03	0.02
CARBHB12	12	6.09	0.02	0.01	0.00	0.00	0.01	0.00	0.00
PNICO23	23	3.49	0.02	0.01	0.01	0.01	0.01	0.01	0.01
HAL59	59	3.90	0.06	0.02	0.01	0.01	0.01	0.01	0.01
AHB21	21	25.40	0.03	0.01	0.01	0.01	0.01	0.01	0.01
CHB6	6	27.04	0.01	0.01	0.01	0.00	0.01	0.01	0.01
IL16	16	110.68	0.08	0.03	0.02	0.01	0.02	0.01	0.01
IDISP	6	18.66	0.59	0.20	0.07	0.03	0.07	0.03	0.02
ICONF	17	3.33	0.01	0.01	0.01	0.01	0.01	0.01	0.01
ACONF	15	2.35	0.06	0.00	0.00	0.00	0.03	0.00	0.00
Amino20x4	80	2.41	0.03	0.01	0.01	0.00	0.01	0.01	0.00
PCONF21	18	2.21	0.10	0.03	0.01	0.01	0.02	0.01	0.01
MCONF	51	4.04	0.13	0.05	0.02	0.00	0.02	0.01	0.01
SCONF	17	4.58	0.03	0.01	0.01	0.01	0.03	0.01	0.01
UPU23	23	6.84	0.12	0.06	0.03	0.01	0.04	0.01	0.01
BUT14DIOL	64	2.93	0.01	0.00	0.00	0.00	0.01	0.01	0.00

5 Extended thermochemistry

Table S5: Statistical error measures for the MOR41 in kcal·mol^{-1}.

	loose	normal	tight	verytight	$l \to n$	$n \to t$	$t \to vt$
MD_C	0.52	0.21	0.09	0.04	0.06	0.04	0.02
MAD_C	0.60	0.26	0.11	0.05	0.10	0.04	0.02
SD_C	0.57	0.28	0.13	0.06	0.17	0.06	0.03
$AMAX_C$	2.09	1.11	0.56	0.26	0.66	0.29	0.11

Table S6: Statistical error measures for the TMCONF16 in kcal·mol^{-1}.

	loose	normal	tight	verytight	$l \to n$	$n \to t$	$t \to vt$
MD_C	-0.04	0.00	-0.01	0.00	0.03	-0.01	0.00
MAD_C	0.04	0.02	0.01	0.00	0.03	0.01	0.00
SD_C	0.04	0.03	0.01	0.00	0.03	0.01	0.01
$AMAX_C$	0.12	0.05	0.02	0.01	0.09	0.03	0.01

Table S7: Statistical error measures for the TMBH in kcal·mol^{-1}.

	loose	normal	tight	verytight	$l \to n$	$n \to t$	$t \to vt$
MD_C	0.11	0.04	0.02	0.01	0.01	0.00	0.00
MAD_C	0.14	0.05	0.02	0.01	0.02	0.01	0.01
SD_C	0.13	0.04	0.02	0.01	0.02	0.01	0.01
$AMAX_C$	0.39	0.14	0.06	0.02	0.04	0.03	0.02

Table S8: Statistical error measures for the ROST61 in kcal·mol^{-1}.

	loose	normal	tight	verytight	$l \to n$	$n \to t$	$t \to$ vt
MD_C	0.24	0.11	0.05	0.02	0.04	0.02	0.01
MAD_C	0.40	0.18	0.09	0.05	0.08	0.05	0.03
SD_C	0.48	0.22	0.11	0.06	0.10	0.06	0.05
$AMAX_C$	1.43	0.65	0.36	0.24	0.30	0.21	0.18

Table S9: Statistical error measures for the TMIP in kcal·mol^{-1}.

	loose	normal	tight	verytight	$l \to n$	$n \to t$	$t \to$ vt
MD_C	0.21	0.10	0.06	0.03	0.04	0.04	0.02
MAD_C	0.58	0.28	0.13	0.06	0.14	0.06	0.03
SD_C	0.76	0.37	0.17	0.08	0.17	0.08	0.04
$AMAX_C$	1.66	0.81	0.35	0.18	0.38	0.21	0.12

Table S10: Statistical error measures for the MOBH35 in kcal·mol^{-1}.

	loose	normal	tight	verytight	$l \to n$	$n \to t$	$t \to$ vt
MD_C	0.19	0.06	0.02	0.01	-0.01	0.00	0.00
MAD_C	0.25	0.09	0.04	0.02	0.04	0.02	0.01
SD_C	0.46	0.16	0.07	0.03	0.05	0.03	0.02
$AMAX_C$	2.04	0.71	0.29	0.13	0.18	0.09	0.06

Table S11: Statistical error measures for the WCCR10 in kcal·mol^{-1}.

	loose	normal	tight	verytight	$l \to n$	$n \to t$	$t \to$ vt
MD_C	-1.12	-0.51	-0.22	-0.09	-0.21	-0.08	-0.02
MAD_C	1.12	0.51	0.22	0.09	0.21	0.10	0.04
SD_C	0.82	0.41	0.22	0.11	0.22	0.14	0.05
$AMAX_C$	2.20	1.07	0.59	0.29	0.54	0.37	0.14

Table S12: Statistical error measures for the IONPI19 in kcal·mol^{-1}.

	loose	normal	tight	verytight	$l \to n$	$n \to t$	$t \to$ vt
MD_C	0.23	0.10	0.04	0.02	0.04	0.01	0.00
MAD_C	0.23	0.10	0.04	0.02	0.04	0.02	0.01
SD_C	0.47	0.22	0.10	0.04	0.09	0.04	0.02
$AMAX_C$	2.08	0.96	0.44	0.18	0.40	0.18	0.05

Table S13: Statistical error measures for the X40x10 in kcal·mol^{-1}.

	loose	normal	tight	verytight	$l \to n$	$n \to t$	$t \to$ vt
MD_C	0.05	0.02	0.01	0.00	0.00	0.00	0.00
MAD_C	0.05	0.02	0.01	0.00	0.01	0.01	0.00
SD_C	0.07	0.02	0.01	0.01	0.01	0.01	0.01
$AMAX_C$	0.48	0.15	0.04	0.03	0.04	0.03	0.04

Table S14: Statistical error measures for the CHAL336 in kcal·mol^{-1}.

	loose	normal	tight	verytight	$l \to n$	$n \to t$	$t \to$ vt
MD_C	0.12	0.05	0.02	0.01	0.01	0.00	0.00
MAD_C	0.12	0.05	0.02	0.01	0.02	0.01	0.01
SD_C	0.09	0.04	0.02	0.02	0.02	0.02	0.02
$AMAX_C$	0.49	0.21	0.11	0.10	0.12	0.10	0.10

Table S15: Statistical error measures for the LP14 in kcal·mol^{-1}.

	loose	normal	tight	verytight	$l \rightarrow n$	$n \rightarrow t$	$t \rightarrow$ vt
MD_C	1.04	0.48	0.23	0.10	0.21	0.11	0.04
MAD_C	1.04	0.48	0.23	0.10	0.16	0.06	0.03
SD_C	0.53	0.28	0.13	0.06	0.55	0.22	0.11
$AMAX_C$	2.14	1.08	0.49	0.21	0.00	0.00	0.00

Table S16: Statistical error measures for the HB300SPX in kcal·mol^{-1}.

	loose	normal	tight	verytight	$l \rightarrow n$	$n \rightarrow t$	$t \rightarrow$ vt
MD_C	0.04	0.02	0.01	0.00	0.00	0.00	0.00
MAD_C	0.04	0.02	0.01	0.01	0.01	0.01	0.01
SD_C	0.03	0.01	0.01	0.01	0.01	0.01	0.01
$AMAX_C$	0.22	0.10	0.06	0.05	0.07	0.06	0.05

Table S17: Statistical error measures for the L7 in kcal·mol^{-1}.

	loose	normal	tight	verytight	$l \rightarrow n$	$n \rightarrow t$	$t \rightarrow$ vt
MD_C	1.34	0.60	0.24	0.08	0.23	0.05	0.00
MAD_C	1.34	0.60	0.24	0.08	0.23	0.08	0.02
SD_C	0.91	0.37	0.13	0.04	0.14	0.07	0.02
$AMAX_C$	2.77	1.23	0.50	0.16	0.46	0.14	0.05

Table S18: Statistical error measures for the ACONFL in kcal·mol^{-1}.

	loose	normal	tight	verytight	$l \rightarrow n$	$n \rightarrow t$	$t \rightarrow$ vt
MD_C	-0.20	-0.07	-0.03	-0.01	-0.01	-0.01	0.00
MAD_C	0.21	0.08	0.04	0.02	0.03	0.01	0.01
SD_C	0.17	0.07	0.03	0.01	0.04	0.01	0.01
$AMAX_C$	0.61	0.16	0.08	0.04	0.09	0.04	0.02

Table S19: Statistical error measures for the S30L in kcal·mol^{-1}.

	loose	normal	tight	verytight	$l \rightarrow n$	$n \rightarrow t$	$t \rightarrow$ vt
MD_C	2.52	1.22	0.60	0.26	0.57	0.28	0.09
MAD_C	2.52	1.22	0.60	0.26	0.57	0.28	0.10
SD_C	1.69	0.80	0.41	0.21	0.45	0.25	0.12
$AMAX_C$	6.41	2.91	1.77	1.02	2.20	1.22	0.64

Table S20: Statistical error measures for the HS13L in kcal·mol^{-1}.

	loose	normal	tight	verytight	$l \to n$	$n \to t$	$t \to$ vt
MD_C	2.29	1.14	0.57	0.28	0.57	0.29	0.13
MAD_C	2.29	1.14	0.57	0.28	0.57	0.29	0.13
SD_C	1.54	0.78	0.41	0.20	0.41	0.24	0.11
$AMAX_C$	6.66	3.29	1.65	0.77	1.61	0.83	0.33

Table S21: Statistical error measures for the revBH9 in kcal·mol^{-1}.

	loose	normal	tight	verytight	$l \to n$	$n \to t$	$t \to$ vt
MD_C	0.29	0.12	0.05	0.04	0.03	0.02	0.04
MAD_C	0.31	0.12	0.05	0.04	0.05	0.02	0.04
SD_C	0.30	0.13	0.06	0.06	0.07	0.03	0.06
$AMAX_C$	1.43	0.76	0.35	0.34	0.43	0.15	0.33

Table S22: Statistical error measures for the revBH9 (reverse) in kcal·mol^{-1}.

	loose	normal	tight	verytight	$l \to n$	$n \to t$	$t \to$ vt
MD_C	0.16	0.06	0.02	0.02	0.02	0.00	0.01
MAD_C	0.19	0.08	0.03	0.02	0.04	0.02	0.03
SD_C	0.23	0.09	0.04	0.04	0.05	0.02	0.05
$AMAX_C$	1.11	0.58	0.24	0.25	0.31	0.11	0.27

Table S23: Statistical error measures for the R160x6 in kcal·mol^{-1}.

	loose	normal	tight	verytight	$l \to n$	$n \to t$	$t \to$ vt
MD_C	0.02	0.01	0.00	0.00	0.00	0.00	0.00
MAD_C	0.02	0.01	0.00	0.00	0.01	0.00	0.00
SD_C	0.03	0.01	0.01	0.01	0.01	0.01	0.01
$AMAX_C$	0.23	0.06	0.02	0.02	0.04	0.03	0.02

6 Geometry benchmark sets

Table S24: Statistical error measures for the CCse21 bond lengths in pm.

	loose	normal	tight	verytight
MD_C	0.001	0.001	0.000	0.000
MAD_C	0.003	0.002	0.002	0.002
SD	0.005	0.003	0.003	0.003
$AMAX_C$	0.017	0.010	0.009	0.009

Table S25: Statistical error measures for the CCse21 angles in °.

	loose	normal	tight	verytight
MD_C	0.000	0.000	0.000	0.000
MAD_C	0.004	0.004	0.004	0.004
SD_C	0.006	0.005	0.005	0.005
$AMAX_C$	0.017	0.013	0.013	0.012

Table S26: Statistical error measures for the HMGB11 bond lengths in pm.

	loose	normal	tight	verytight
MD_C	0.055	0.019	0.006	0.001
MAD_C	0.055	0.019	0.006	0.004
SD_C	0.041	0.018	0.008	0.006
$AMAX_C$	0.117	0.045	0.021	0.014

Table S27: Statistical error measures for the TMC32 bond lengths in pm.

	loose	normal	tight	verytight
MD_C	0.047	0.033	0.020	0.015
MAD_C	0.298	0.070	0.035	0.024
SD_C	0.871	0.104	0.072	0.053
$AMAX_C$	4.472	0.399	0.423	0.309

Table S28: Statistical error measures for the ROT34 rotational constants in MHz.

	loose	normal	tight	verytight
MD_C	-0.379	-0.129	-0.050	-0.009
MAD_C	0.526	0.224	0.144	0.121
SD_C	0.632	0.280	0.185	0.164
$AMAX_C$	2.500	0.900	0.500	0.400

Table S29: Statistical error measures for the LMGB bond lengths in pm.

	loose	normal	tight	verytight
MD_C	0.000	0.000	0.000	0.000
MAD_C	0.003	0.003	0.003	0.003
SD_C	0.005	0.005	0.005	0.005
$AMAX_C$	0.017	0.017	0.017	0.017

Table S30: Statistical error measures for the LB12 bond lengths in pm.

	loose	normal	tight	verytight
MD_C	0.689	0.323	0.152	0.059
MAD_C	0.701	0.339	0.164	0.083
SD_C	0.900	0.416	0.213	0.116
$AMAX_C$	2.418	1.039	0.623	0.306
